# Dynamic serum tumor-marker and immune-inflammatory biomarker changes for risk stratification of immune checkpoint inhibitor-associated myasthenia gravis in non-small cell lung cancer

**DOI:** 10.3389/fimmu.2026.1929526

**Published:** 2026-09-10

**Authors:** Pin Cui, Xiaohui Lin, Weimin Luo, Shaorong Zhang, Song Zhang, Shu Xu, Junming Lai, Yong Xiong, Dehong Chen, Binding Huang, Zhiming Xu

**Affiliations:** 1 Department of Geriatric Orthopedics, Sichuan Provincial Orthopedic Hospital Shenzhen Rapha Biotechnology Incorporate, Shenzhen, China; 2 Department of Oncology, People’s Hospital of Shenzhen Baoan District, The Second Affiliated Hospital of Shenzhen University, Shenzhen, China; 3 Department of Cardiothoracic Surgery, Shenzhen University of Advanced Technology General Hospital, Shenzhen, China; 4 Department of Gastroenterology, Shenzhen University of Advanced Technology General Hospital, Shenzhen, China; 5 Department of Otolaryngology, Shenzhen University of Advanced Technology General Hospital, Shenzhen, China; 6 Cancer Center, Shenzhen University of Advanced Technology General Hospital, Shenzhen, China; 7 Department of Urologic Surgery, Shenzhen University of Advanced Technology General Hospital, Shenzhen, China; 8 Department of Gastrointestinal Surgery, Shenzhen University of Advanced Technology General Hospital, Shenzhen, China; 9 College of Artificial Intelligence, Shenzhen Technology University, Shenzhen, China

**Keywords:** albumin, biomarker dynamics, C-reactive protein, immune checkpoint inhibitor, immune-related adverse event, lactate dehydrogenase, myasthenia gravis, neutrophil-to-lymphocyte ratio

## Abstract

**Background:**

Immune checkpoint inhibitors (ICIs) have reshaped the treatment landscape of non-small cell lung cancer (NSCLC), but immune-related neuromuscular toxicities remain difficult to anticipate. ICI-associated myasthenia gravis is uncommon, frequently occurs early after treatment initiation, and may overlap with myositis or myocarditis. Clinically accessible blood-based indicators that help identify patients entering this high-risk period remain insufficiently defined.

**Objective:**

This study evaluated whether early dynamic changes in serum tumor markers and immune-inflammatory biomarkers could serve as noninvasive indicators of subsequent ICI-associated myasthenia gravis in patients with NSCLC.

**Methods:**

Consecutive patients with NSCLC who received their first ICI-based therapy between 1 January 2018 and 31 January 2025 were retrospectively screened at a single center. Baseline, Day-21, and Day-42 laboratory windows were prespecified before endpoint modeling. Serum tumor markers included carcinoembryonic antigen, cytokeratin 19 fragment antigen 21-1, neuron-specific enolase, squamous cell carcinoma antigen, and carbohydrate antigen 125. Immune-inflammatory biomarkers included neutrophil-to-lymphocyte ratio, platelet-to-lymphocyte ratio, systemic immune-inflammation index, systemic inflammation response index, C-reactive protein, lactate dehydrogenase, and albumin. The primary endpoint was definite or probable ICI-associated myasthenia gravis occurring within 180 days after first ICI exposure. Landmark models were developed using Firth bias-reduced logistic regression and internally validated by bootstrap resampling. The Day-21 dynamic score used clinically pragmatic thresholds defined before endpoint modeling.

**Results:**

The final baseline cohort included 826 patients, of whom 27 developed definite or probable ICI-associated myasthenia gravis within 180 days. The Day-21 landmark cohort included 782 patients and 21 events occurring after Day 21. Compared with non-event patients, patients who later developed ICI-associated myasthenia gravis showed greater Day-21 increases in cytokeratin 19 fragment antigen 21-1, carcinoembryonic antigen, neutrophil-to-lymphocyte ratio, C-reactive protein, and lactate dehydrogenase, together with a larger decline in albumin. A four-component Day-21 dynamic score combining tumor-marker instability, neutrophil-to-lymphocyte ratio activation, C-reactive protein/lactate dehydrogenase flare, and albumin decline stratified event rates after Day 21 from 1.2% in the low-risk group to 6.7% in the high-risk group. The integrated Day-21 model achieved an apparent area under the receiver operating characteristic curve of 0.759 and an optimism-corrected area under the receiver operating characteristic curve of 0.731, indicating moderate internal discrimination.

**Conclusion:**

Early dynamic changes in routine serum tumor markers and immune-inflammatory biomarkers were associated with increased risk of ICI-associated myasthenia gravis in patients with NSCLC. The Day-21 integrated biomarker score may help identify patients requiring closer neuromuscular and cardiac toxicity surveillance during the first few weeks of ICI therapy, but external validation is required before clinical implementation.

## 1 Introduction

Lung cancer remains a major cause of cancer morbidity and mortality worldwide, and non-small cell lung cancer (NSCLC) accounts for most lung cancer cases requiring systemic treatment (1). Immune checkpoint inhibitors (ICIs) targeting programmed cell death protein 1, programmed death-ligand 1, or cytotoxic T-lymphocyte-associated protein 4 have become central to the management of advanced NSCLC, either as monotherapy or in combination with chemotherapy, antiangiogenic therapy, or other immune agents (2). By releasing inhibitory immune checkpoints, these treatments can produce durable antitumor responses, but the same immune activation can also disrupt tissue tolerance and cause immune-related adverse events involving multiple organs (3).

In NSCLC, immune-related adverse events have a complex clinical meaning. Their occurrence may reflect immune activation and has been associated with favorable treatment outcomes in several cohorts, but this association does not lessen the risk posed by severe neurologic, cardiac, or overlapping toxicities (4). ICI-associated myasthenia gravis is particularly challenging because it is uncommon, may begin with nonspecific fatigue or ocular symptoms, and can progress rapidly after ICI exposure (5). Neuromuscular immune-related adverse events may also present as mixed syndromes involving myasthenia gravis, inflammatory myopathy, and peripheral neuropathic features, making early recognition more difficult than recognition of many cutaneous or endocrine toxicities (6, 7).

The clinical importance of ICI-associated myasthenia gravis is amplified by its overlap with myositis and myocarditis. Reported cohorts and systematic reviews have shown that patients with ICI-associated myasthenia gravis may develop severe weakness, respiratory compromise, concurrent muscle injury, or cardiac involvement, and these overlap syndromes account for a disproportionate share of adverse outcomes (8). Myocarditis–myositis–myasthenia gravis overlap is therefore a high-risk phenotype in which delayed recognition may lead to intensive care admission, ventilatory support, or death (9). A practical early warning strategy should not rely solely on confirmatory neuromuscular tests, because antibody testing, electrophysiology, creatine kinase, and troponin are often ordered after symptoms have already emerged.

Routine blood biomarkers may help narrow this early risk window, although most are not specific to myasthenia gravis. In advanced NSCLC treated with PD-1 or PD-L1 inhibitors, early changes in serum tumor markers such as carcinoembryonic antigen and cytokeratin 19 fragment antigen 21–1 have been associated with treatment response and survival, suggesting that dynamic tumor-marker behavior captures part of the early tumor–host interaction after ICI initiation (10). Peripheral blood inflammatory markers also provide accessible information on host immune status; the neutrophil-to-lymphocyte ratio and related blood-cell indices have been linked to immune-related toxicity risk in advanced NSCLC treated with checkpoint inhibitors (11). However, tumor-marker increases may reflect tumor burden, delayed response, pseudoprogression, tissue injury, or systemic immune activation, while C-reactive protein, lactate dehydrogenase, neutrophil-to-lymphocyte ratio, systemic immune-inflammation index, and systemic inflammation response index are acute-phase indicators rather than markers specific to the neuromuscular junction. Existing studies have mostly evaluated broad immune-related adverse events or oncologic outcomes rather than ICI-associated myasthenia gravis as a specific neuromuscular endpoint.

The premise of this study was that early post-ICI instability in tumor-marker and immune-inflammatory profiles may identify a broader tumor–host immune activation state in which neuromuscular, myopathic, or cardiac immune-related toxicities are more likely to emerge. This study therefore sought to establish a clinically reproducible data chain linking early serum tumor-marker dynamics and immune-inflammatory biomarker changes with subsequent ICI-associated myasthenia gravis in NSCLC. We focused on routinely available measurements at baseline, Day 21, and Day 42, excluded post-symptom diagnostic markers from the primary prediction model, and used landmark analysis to reduce information leakage. The aim was not to replace neurologic adjudication, but to determine whether early noninvasive biomarker changes could identify patients requiring closer surveillance for ocular, bulbar, respiratory, myopathic, and cardiac overlap features during the first few weeks of ICI therapy. Given the single-center retrospective design and the small number of endpoint events, the resulting model was intended for exploratory early risk stratification and requires external validation.

## 2 Materials and methods

### 2.1 Study design and ethics

**Figure 1:**
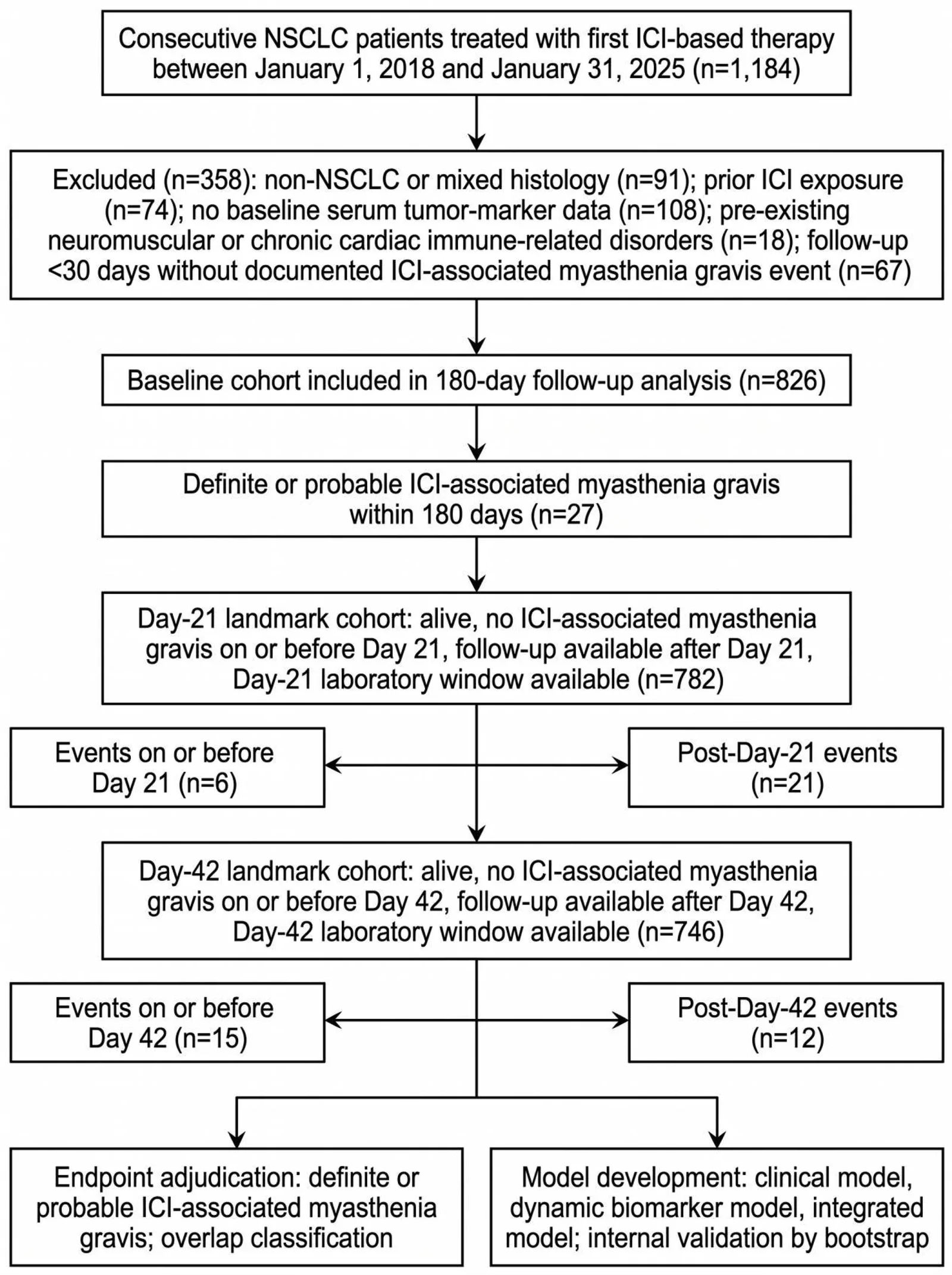
Study workflow.

This single-center retrospective cohort study was conducted at the Second Affiliated Hospital of Shenzhen University. Consecutive patients with non-small cell lung cancer (NSCLC) who received at least one dose of immune checkpoint inhibitor (ICI)-based therapy between 1 January 2018 and 31 January 2025 were screened, and follow-up was censored on 31 July 2025. The study followed the STROBE statement for observational cohort studies and the TRIPOD statement for prediction model reporting (12, 13). The protocol was approved by the Ethics Committee of the Second Affiliated Hospital of Shenzhen University (Protocol No. SZB9281X). De-identified data were extracted from routine clinical records without additional blood sampling, imaging, treatment assignment, patient contact, or study-specific intervention, and the requirement for informed consent was waived by the ethics committee because of the retrospective, de-identified design. The cohort-screening process, laboratory landmark windows, endpoint adjudication, and model-development workflow are shown in **Figure 1**.

### 2.2 Study population

Patients were eligible if they were 18 years or older at ICI initiation, had pathologically confirmed NSCLC, had unresectable stage III, stage IV, or postoperative recurrent disease, received their first exposure to a PD-1 inhibitor, PD-L1 inhibitor, CTLA-4 inhibitor, or combined ICI regimen, and had at least one baseline complete blood count plus at least one baseline serum tumor marker measured within 7 days before the first ICI infusion. Histology was recorded as adenocarcinoma, squamous cell carcinoma, large-cell carcinoma, or NSCLC not otherwise specified according to the 2021 World Health Organization thoracic tumor classification, and stage was recorded according to the eighth edition TNM classification for lung cancer (14, 15).

Patients were excluded if they had small-cell lung cancer, combined small-cell lung cancer, pulmonary neuroendocrine carcinoma other than large-cell neuroendocrine carcinoma, metastatic lung involvement from another primary tumor, prior ICI exposure, preexisting myasthenia gravis, Lambert–Eaton myasthenic syndrome, inflammatory myopathy, muscular dystrophy, motor neuron disease, chronic myocarditis, active autoimmune neuromuscular disease, blinded trial therapy without accessible ICI class, follow-up shorter than 30 days without a documented ICI-associated myasthenia gravis event, or insufficient source records for endpoint adjudication. The index date was the date of the first ICI administration. The baseline cohort was used for cohort description and ascertainment of cumulative 180-day endpoints. The Day-21 landmark cohort included patients who were alive, had no definite or probable ICI-associated myasthenia gravis before or on Day 21, had at least one follow-up record after Day 21, and had available laboratory data within the Day-21 window. The Day-42 landmark cohort was defined using the same criteria at Day 42. Patients who were excluded from the Day-21 landmark cohort because of missing Day-21 laboratory data or unavailable post-landmark follow-up were compared with included patients using baseline characteristics and standardized mean differences.

### 2.3 Treatment exposure and baseline variables

ICI exposure was extracted from medication administration records. PD-1 inhibitors included nivolumab, pembrolizumab, camrelizumab, sintilimab, tislelizumab, toripalimab, and other documented PD-1 antibodies. PD-L1 inhibitors included atezolizumab, durvalumab, and sugemalimab. CTLA-4-containing therapy included ipilimumab or another documented CTLA-4 antibody. Treatment regimens were categorized as ICI monotherapy, ICI plus platinum-based chemotherapy, ICI plus antiangiogenic therapy, ICI plus chemotherapy and antiangiogenic therapy, or dual ICI therapy, and the distribution of regimens was compared between patients with and without post-landmark ICI-associated myasthenia gravis.

Baseline variables included age at ICI initiation, sex, smoking history, body mass index, Eastern Cooperative Oncology Group performance status, histological subtype, disease stage, treatment line, ICI class, combination regimen, programmed death-ligand 1 tumor proportion score when available, epidermal growth factor receptor mutation status, anaplastic lymphoma kinase rearrangement status, liver metastasis, brain metastasis, prior thoracic radiotherapy, prior systemic chemotherapy, prior targeted therapy, baseline autoimmune disease, chronic obstructive pulmonary disease, diabetes mellitus, coronary artery disease, chronic kidney disease, chronic liver disease, and systemic corticosteroid exposure within 14 days before ICI initiation. Baseline systemic corticosteroid exposure was defined as a prednisone-equivalent dose of at least 10 mg/day for 7 or more consecutive days before ICI initiation; inhaled, topical, intra-articular, and single-dose antiemetic corticosteroid use was not counted. Radiographic response was recorded according to RECIST version 1.1 when measurable disease and scheduled imaging were available, but it was not used as a primary predictor because response assessment usually occurred after the Day-21 biomarker window (16). Best overall response within 180 days and non-myasthenic immune-related adverse events were retained for exploratory descriptive analyses, but they were not entered into the primary Day-21 prediction model.

### 2.4 Laboratory measurement windows and assay variables

Laboratory data were extracted from the hospital laboratory information system, and only clinically released venous blood results processed by the central hospital laboratory were used. Baseline values were defined as the result closest to the first ICI administration within −7 to 0 days. If same-day testing and ICI infusion occurred on the index date, the pre-infusion value was used. Day-21 values were defined as the result closest to Day 21 within Days 14–28 after the first ICI administration, and Day-42 values were defined as the result closest to Day 42 within Days 35–49. When more than one result was available in a window, the result closest to the target day was selected; if two results were equally close, the earlier result was selected. Laboratory values measured after the first documented symptom of suspected ICI-associated myasthenia gravis were not used in the corresponding landmark model.

Serum tumor markers included carcinoembryonic antigen (CEA), cytokeratin 19 fragment antigen 21-1 (CYFRA21-1), neuron-specific enolase (NSE), squamous cell carcinoma antigen (SCC-Ag), and carbohydrate antigen 125 (CA125). CEA and CYFRA21–1 were treated as the primary tumor-marker variables because early changes in these routinely available markers have been evaluated in advanced NSCLC treated with PD-1 or PD-L1 inhibitors (17–19). Immune-inflammatory biomarkers included absolute neutrophil count, absolute lymphocyte count, absolute monocyte count, platelet count, C-reactive protein (CRP), lactate dehydrogenase (LDH), and serum albumin. Neutrophil-to-lymphocyte ratio (NLR) was calculated as absolute neutrophil count divided by absolute lymphocyte count. Platelet-to-lymphocyte ratio (PLR) was calculated as platelet count divided by absolute lymphocyte count. Systemic immune-inflammation index (SII) was calculated as platelet count multiplied by absolute neutrophil count and divided by absolute lymphocyte count. Systemic inflammation response index (SIRI) was calculated as absolute neutrophil count multiplied by absolute monocyte count, divided by absolute lymphocyte count. All measurement windows, variables, and analytic roles were prespecified before endpoint modeling, as summarized in **Table 1** (20, 21). Dynamic-score thresholds are described in *Section 2.5*.

**Table 1:** Measurement windows and derived variables.

Variable group	Variables	Measurement window	Analytic role or derived variable
Baseline clinical data	Age, sex, smoking status, body mass index, ECOG performance status, histology, stage, treatment line, ICI regimen, comorbidities	Closest eligible record before first ICI dose	Retained as baseline covariates
Serum tumor markers	CEA, CYFRA21-1, NSE, SCC-Ag, CA125	Baseline: −7 to 0 days; Day 21: Days 14–28; Day 42: Days 35–49	Absolute value and Percentage change from baseline
Blood-cell indices	Absolute neutrophil count, absolute lymphocyte count, absolute monocyte count, platelet count	Same as serum tumor-marker windows	NLR, PLR, SII, and SIRI; absolute value and percentage change from baseline
Inflammatory biochemistry	CRP, LDH, albumin	Same as serum tumor-marker windows	Absolute value and absolute change from baseline
Neuromuscular and cardiac injury markers	Creatine kinase, creatine kinase-MB, cardiac troponin, NT-proBNP or BNP	Extracted when clinically ordered	Used for endpoint adjudication and overlap classification only; not used as primary predictors
Treatment exposure	ICI class, combination regimen, treatment line, baseline systemic corticosteroid exposure	Baseline to landmark date	Retained as treatment covariates when prespecified
Tumor response and non-myasthenic immune-related adverse events	RECIST best overall response and immune-related adverse events other than ICI-associated myasthenia gravis	First ICI dose to 180 days	Used for exploratory descriptive analyses; not used as primary predictors
Follow-up and severity data	Neurology consultation, cardiology consultation, intensive care admission, ventilatory support, intravenous immunoglobulin, plasma exchange, death	First ICI dose to 180 days	Used for endpoint and severity adjudication

BNP, B-type natriuretic peptide; CA125, carbohydrate antigen 125; CEA, carcinoembryonic antigen; CRP, C-reactive protein; CYFRA21-1, cytokeratin 19 fragment antigen 21-1; ECOG, Eastern Cooperative Oncology Group; ICI, immune checkpoint inhibitor; LDH, lactate dehydrogenase; NLR, neutrophil-to-lymphocyte ratio; NSE, neuron-specific enolase; NT-proBNP, N-terminal pro-B-type natriuretic peptide; PLR, platelet-to-lymphocyte ratio; RECIST, Response Evaluation Criteria in Solid Tumors; SCC-Ag, squamous-cell carcinoma antigen; SII, systemic immune-inflammation index; SIRI, systemic inflammation response index.

### 2.5 Dynamic biomarker definitions

Percentage change was calculated as (follow-up value − baseline value) divided by baseline value and multiplied by 100. Absolute change was calculated as follow-up value minus baseline value. CEA, CYFRA21-1, NSE, SCC-Ag, CA125, CRP, LDH, NLR, PLR, SII, and SIRI were natural-log transformed using ln(value + 1) for continuous modeling when right-skewed distributions were present.

The primary Day-21 dynamic biomarker score included four components. One point was assigned for tumor-marker instability, defined as a CYFRA21–1 increase of at least 20% or a CEA increase of at least 25% from baseline to Day 21. One point was assigned for NLR activation, defined as a Day-21 NLR of at least 5.0 or an NLR increase of at least 50% from baseline. One point was assigned for inflammatory flare, defined as a CRP increase of at least 10 mg/L or an LDH increase of at least 40 U/L from baseline. One point was assigned for albumin decline, defined as a serum albumin decrease of at least 3 g/L from baseline to Day 21. The score ranged from 0 to 4 points and was categorized as low risk (0 points), intermediate risk (1 point), and high risk (2 or more points). The same scoring structure was applied to the Day-42 analysis. These clinically pragmatic thresholds were defined before endpoint modeling and were not selected by receiver operating characteristic analysis, Youden index optimization, or endpoint-driven maximization in this cohort. The four components were selected to represent tumor-marker instability, neutrophil-predominant inflammatory activation, acute-phase inflammatory or tissue-injury flare, and albumin decline. PLR, SII, and SIRI were retained as secondary continuous-change biomarkers rather than categorical score components because they overlapped mathematically with complete-blood-count-derived inflammatory indices and would have increased the score complexity relative to the number of endpoint events.

Threshold-perturbation sensitivity analyses varied the thresholds for CYFRA21–1 increase at 15%, 20%, and 25%; CEA increase at 20%, 25%, and 30%; Day-21 NLR at ≥4.0, ≥5.0, and ≥6.0; NLR increase at ≥30%, ≥50%, and ≥70%; CRP increase at ≥5, ≥10, and ≥15 mg/L; LDH increase at ≥30 U/L, ≥40 U/L, and ≥50 U/L; and albumin decline at ≥2 g/L, ≥3 g/L, and ≥4 g/L. A continuous-change model using ln-transformed Day-21 biomarker changes was also fitted to compare threshold-based and non-threshold-based modeling.

Acute infection within a biomarker window was defined as clinician-documented infection requiring systemic antimicrobial therapy, with or without positive microbiological culture. Patients with acute infection during the biomarker window were retained in the primary analysis and excluded in a prespecified sensitivity analysis.

### 2.6 Identification of suspected neuromuscular immune-related adverse events

Potential ICI-associated myasthenia gravis cases were identified using diagnosis codes, diagnostic terms, consultation notes, laboratory triggers, medication records, and treatment-interruption records. Trigger terms included myasthenia gravis, neuromuscular junction disorder, immune-related neuromuscular toxicity, myositis, myocarditis, ptosis, diplopia, dysphagia, dysarthria, neck weakness, generalized fatigable weakness, respiratory muscle weakness, unexplained respiratory failure, and immune-related adverse event. Trigger tests and treatments included acetylcholine receptor antibody, muscle-specific kinase antibody, repetitive nerve stimulation, single-fiber electromyography, needle electromyography, pyridostigmine, intravenous immunoglobulin, plasma exchange, and high-dose corticosteroid therapy for suspected neuromuscular immune toxicity. Creatine kinase elevation with weakness or myalgia, cardiac troponin elevation with neuromuscular symptoms, neurology consultation after ICI initiation, and ICI interruption because of suspected neuromuscular toxicity also triggered manual review. Trigger-positive records without ocular, bulbar, respiratory, axial, or limb weakness after ICI exposure were classified as non-cases after source-record review. All trigger-positive suspected cases, rather than only final positive cases, formed the denominator for reviewer-agreement assessment.

### 2.7 Endpoint adjudication

The primary endpoint was definite or probable ICI-associated myasthenia gravis within 180 days after the first ICI exposure. Endpoint grading followed CTCAE version 5.0, and clinical severity was described using the Myasthenia Gravis Foundation of America clinical classification when sufficient neurologic documentation was available (22, 23). Two clinicians independently reviewed all suspected cases while blinded to the Day-21 and Day-42 dynamic biomarker scores. Initial interreviewer agreement was calculated across all trigger-positive suspected cases using a 2 × 2 agreement matrix and Cohen κ. A definite case required new fatigable ocular, bulbar, respiratory, axial, or limb weakness after ICI exposure; neurology or oncology documentation indicating suspected myasthenia gravis or a neuromuscular junction disorder; at least one supportive feature, including a positive acetylcholine receptor antibody, a positive muscle-specific kinase antibody, abnormal repetitive nerve stimulation, abnormal single-fiber electromyography, a compatible specialist neuromuscular examination, or a clear clinical response to myasthenia gravis-directed therapy; and no stronger alternative diagnosis.

A probable case required new ocular, bulbar, respiratory, axial, or generalized fatigable weakness after ICI exposure, clinical management as immune-related myasthenia gravis, and no stronger alternative diagnosis, even when antibody or electrophysiologic confirmation was unavailable. Antibody negativity did not exclude ICI-associated myasthenia gravis. Disagreement was resolved by a third senior reviewer. Time to onset was calculated from the first ICI administration to the first documented neuromuscular symptom attributable to ICI-associated myasthenia gravis. Myositis overlap was defined as definite or probable ICI-associated myasthenia gravis plus myalgia or proximal weakness with creatine kinase above five times the institutional upper reference limit, or electromyography, muscle magnetic resonance imaging, or biopsy findings supporting inflammatory myopathy. Myocarditis overlap was defined as definite or probable ICI-associated myasthenia gravis plus cardiac troponin above the institutional upper reference limit and at least one supportive feature, including a new electrocardiographic abnormality, new ventricular dysfunction, cardiac magnetic resonance imaging compatible with myocarditis, endomyocardial biopsy, or a cardiology diagnosis after exclusion of acute coronary syndrome when clinically indicated. Myocarditis–myositis–myasthenia gravis overlap was defined when all three domains were present within the same immune-related episode. The endpoint-adjudication rules are summarized in **Table 2** (24, 25).

**Table 2:** Endpoint adjudication rules.

Endpoint	Operational definition	Required evidence
Definite ICI-associated myasthenia gravis	New fatigable ocular, bulbar, respiratory, axial, or limb weakness after ICI exposure plus supportive antibody, electrophysiological, examination, or treatment-response evidence	Neurology or oncology documentation plus laboratory, electrophysiology, medication, or examination support
Probable ICI-associated myasthenia gravis	Clinically treated ICI-related myasthenia gravis with a typical symptom pattern and no stronger alternative explanation	Specialist documentation, treatment record, and exclusion of competing causes
ICI-associated myasthenia gravis with myositis	Definite or probable ICI-associated myasthenia gravis plus myalgia or proximal weakness with creatine kinase >5 times the institutional upper reference limit, or electromyography, muscle MRI, or biopsy support	Creatine kinase record plus clinical or instrumental evidence
ICI-associated myasthenia gravis with myocarditis	Definite or probable ICI-associated myasthenia gravis plus cardiac troponin above the institutional upper reference limit and supportive cardiac evidence	Troponin record plus electrocardiography, echocardiography, cardiac MRI, biopsy, or cardiology diagnosis
Myocarditis–myositis–myasthenia gravis overlap	ICI-associated myasthenia gravis, myositis, and myocarditis in the same immune-related episode	All three endpoint domains fulfilled
Severe ICI-associated myasthenia gravis outcome	Intensive care admission, ventilatory support, intravenous immunoglobulin, plasma exchange, or death attributable to ICI-associated myasthenia gravis or overlap syndrome	Intensive care record, respiratory support record, treatment record, discharge summary, or death record

ICI, immune checkpoint inhibitor; MRI, magnetic resonance imaging.

### 2.8 Confounding control and information-leakage boundaries

Only variables available before or within the relevant landmark window were eligible as predictors. Variables occurring after the landmark time point were not used in the corresponding prediction model. Creatine kinase, creatine kinase-MB, cardiac troponin, NT-proBNP or BNP, acetylcholine receptor antibody, muscle-specific kinase antibody, electrophysiologic findings, pyridostigmine initiation, intravenous immunoglobulin, plasma exchange, high-dose corticosteroid therapy after symptom onset, intensive care admission, ventilatory support, and ICI interruption due to suspected toxicity were not used as primary predictors because these variables were part of endpoint recognition, overlap classification, or post-event management.

Prespecified confounders included age, sex, smoking status, Eastern Cooperative Oncology Group performance status, histology, stage, treatment line, ICI regimen category, baseline autoimmune disease, baseline systemic corticosteroid exposure, chronic obstructive pulmonary disease, coronary artery disease, diabetes mellitus, chronic kidney disease, liver metastasis, brain metastasis, and acute infection during the biomarker window. Programmed death-ligand 1 tumor proportion score, epidermal growth factor receptor mutation status, and anaplastic lymphoma kinase rearrangement status were retained for descriptive analysis and sensitivity analyses when missingness allowed. Post-baseline radiographic response, ICI discontinuation, dose delay after suspected toxicity, emergency department visit after neuromuscular symptoms, and hospitalization for suspected toxicity were not included as baseline or landmark predictors. Best overall tumor response and non-myasthenic immune-related adverse events were analyzed descriptively to contextualize biomarker changes but were not used for primary risk-score construction or model fitting. Death before ICI-associated myasthenia gravis was treated as a competing clinical event in the sensitivity analysis.

### 2.9 Data quality control and missing data

Data were extracted from the electronic medical record, oncology treatment system, laboratory information system, radiology reporting system, pharmacy record, discharge summary, intensive care record, and consultation notes. A structured data dictionary was completed before analysis. All suspected ICI-associated myasthenia gravis cases and a random 10% sample of non-cases were checked against source records. Duplicate laboratory values, impossible dates, incompatible units, and inconsistent treatment dates were resolved by returning to the original clinical report. Laboratory values below the lower reporting limit were assigned half of the lower reporting limit for continuous analysis. Values above the analytical range were retained only when a verified diluted or final released value was available; otherwise, they were treated as missing. Continuous laboratory variables were winsorized at the 1st and 99th percentiles after source verification.

Variables with more than 20% missingness were excluded from the primary multivariable modeling. Variables with 5% to 20% missingness were handled using multiple imputation by chained equations with 20 imputed datasets. The imputation model included endpoint status, candidate predictors, treatment variables, follow-up time, and auxiliary variables related to missingness. Variables with less than 5% missingness were analyzed using complete available records in the primary analysis and checked using imputation in the sensitivity analysis. Endpoint status was not imputed (26). Patients without an eligible Day-21 laboratory window or without post-Day-21 follow-up were excluded from the Day-21 landmark cohort and compared with included patients to evaluate potential attrition-related selection bias.

### 2.10 Statistical analysis

Continuous variables were summarized as means with standard deviations or medians with interquartile ranges, according to distribution. Categorical variables were summarized as frequencies and percentages. Baseline imbalance was described using standardized mean differences rather than significance testing because the endpoint was uncommon. The primary analysis evaluated post-Day-21 ICI-associated myasthenia gravis in the Day-21 landmark cohort, and the secondary analysis evaluated post-Day-42 ICI-associated myasthenia gravis in the Day-42 landmark cohort.

Three prespecified models were developed. The clinical model included age, sex, Eastern Cooperative Oncology Group performance status, ICI regimen category, baseline autoimmune disease, and baseline systemic corticosteroid exposure. The biomarker model included the four-component dynamic biomarker score. The integrated model combined the clinical variables and the dynamic biomarker score. Firth bias-reduced logistic regression was used for the primary multivariable modeling because the endpoint was uncommon and separation could occur in conventional logistic regression (27). Model discrimination was assessed using the area under the receiver operating characteristic curve and the area under the precision–recall curve. Calibration was assessed using the calibration intercept, calibration slope, Brier score, and calibration plots. Internal validation was performed using 1,000 bootstrap resamples to estimate optimism-corrected performance. Within each bootstrap resample, Day-21 score components were recalculated from baseline and Day-21 biomarker values, score categories were reassigned, the Firth model was refitted, and discrimination and calibration were re-estimated. Because the primary thresholds were defined before endpoint modeling, no receiver operating characteristic-derived cutoff selection was performed inside or outside the bootstrap procedure. Clinical usefulness was evaluated using decision-curve analysis across threshold probabilities from 1% to 10% (28).

Prespecified sensitivity analyses included the exclusion of patients with acute infection during the biomarker window; the exclusion of patients receiving baseline systemic corticosteroids; restriction to PD-1 or PD-L1 monotherapy and ICI plus chemotherapy; restriction to definite ICI-associated myasthenia gravis; use of ICI-associated myasthenia gravis with myositis or myocarditis overlap as a composite severe neuromuscular endpoint; replacement of the categorical four-component score with continuous Day-21 biomarker changes; threshold-perturbation analysis of the four-component score; repetition using the Day-42 landmark cohort; and competing-risk sensitivity analysis treating death before ICI-associated myasthenia gravis as a competing clinical event. Exploratory analyses compared the distribution of treatment regimens, best overall tumor response, and non-myasthenic immune-related adverse events across Day-21 risk strata. All analyses were performed using R version 4.4.2. The primary R packages were tidyverse for data management, survival for time-to-event summaries, mice for multiple imputation, logistf or brglm2 for bias-reduced logistic regression, pROC for receiver operating characteristic analysis, PRROC for precision–recall analysis, rms for calibration and bootstrap validation, and rmda or dcurves for decision-curve analysis. A two-sided P value of less than 0.05 was considered statistically significant.

## 3 Results

### 3.1 Cohort construction, landmark sets, and endpoint distribution

**Figure 2:**
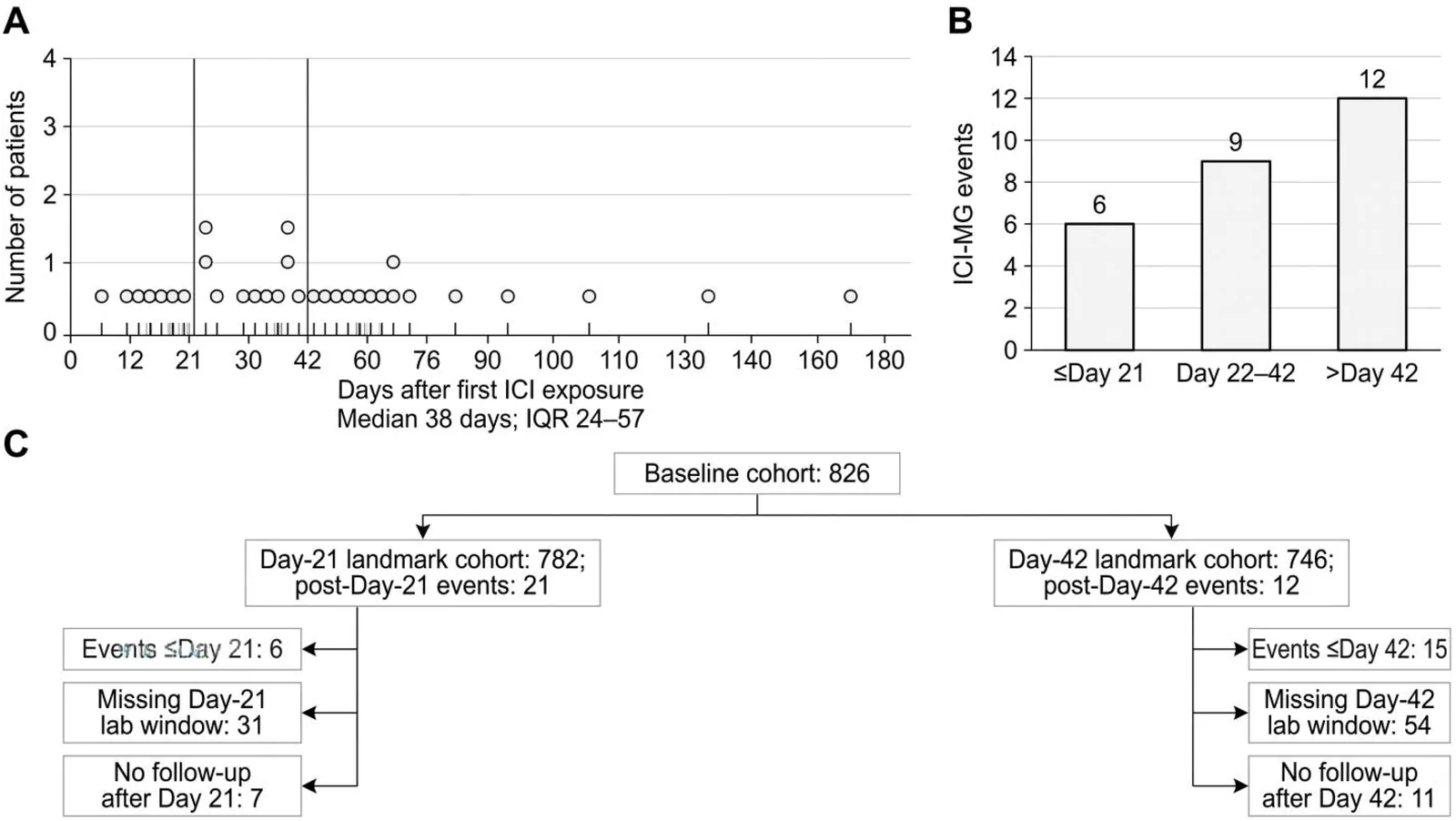
Endpoint timing after the first ICI exposure. **(A)** Distribution of time from the first ICI administration to first documented neuromuscular symptom among patients with definite or probable ICI-associated myasthenia gravis. **(B)** Event counts before or on Day 21, between Days 22 and 42, and after Day 42. **(C)** Landmark-cohort event distribution for the Day-21 and Day-42 analyses.

Among 1,184 screened patients, 826 entered the baseline cohort after exclusion of 91 patients with non-NSCLC or mixed histology, 74 with prior ICI exposure, 108 without baseline serum tumor-marker data, 18 with preexisting neuromuscular or chronic cardiac immune-related disorders, and 67 with follow-up shorter than 30 days without a documented ICI-associated myasthenia gravis event. Within 180 days after the first ICI exposure, 27 patients developed definite or probable ICI-associated myasthenia gravis, corresponding to a cumulative 180-day event proportion of 3.3%. Six events occurred before or on Day 21. Among the remaining patients eligible for Day-21 landmark assessment, 31 lacked an available Day-21 laboratory window and 7 had no evaluable follow-up after Day 21, leaving 782 patients in the Day-21 landmark cohort, with 21 post-Day-21 events. Fifteen events occurred before or on Day 42. Among patients eligible for Day-42 landmark assessment, 54 lacked an available Day-42 laboratory window and 11 had no evaluable follow-up after Day 42, leaving 746 patients in the Day-42 landmark cohort, with 12 post-Day-42 events. The median time from the first ICI administration to the first neuromuscular symptom was 38 days (interquartile range, 24–57), and 15 of 27 events occurred within the first 42 days after ICI initiation. Endpoint timing is shown in **Figure 2**.

Core baseline clinical variables had low missingness in the Day-21 landmark cohort. Missingness was 0% for age, sex, treatment regimen, and endpoint status; 1.2% for body mass index; 2.0% for ECOG performance status; 16.4% for programmed death-ligand 1 tumor proportion score; 5.4% for epidermal growth factor receptor mutation status; and 6.5% for anaplastic lymphoma kinase rearrangement status. Variables with missingness above 20% were not entered into the primary multivariable models. Patients included in the Day-21 landmark cohort and patients excluded because of missing Day-21 laboratory data or unavailable post-landmark follow-up showed no large baseline imbalance; all standardized mean differences were below 0.20, except for baseline systemic corticosteroid exposure, which remained uncommon in both groups (**Table 3**).

**Table 3:** Baseline comparison of patients included in and excluded from the Day-21 landmark cohort.

Characteristic	Included in day-21 landmark cohort *n* = 782	Excluded from day-21 landmark cohort *n* = 38	Standardized mean difference
Age, years	65.5 ± 9.2	66.8 ± 9.5	0.14
Male sex	529 (67.6%)	27 (71.1%)	0.08
Current or former smoker	536 (68.5%)	27 (71.1%)	0.05
Body mass index, kg/m²	23.1 ± 3.4	22.8 ± 3.6	0.09
ECOG performance status ≥2	123 (15.7%)	8 (21.1%)	0.14
Stage IV or recurrent disease	641 (82.0%)	32 (84.2%)	0.06
First-line ICI therapy	461 (58.9%)	21 (55.3%)	0.07
ICI monotherapy	238 (30.4%)	12 (31.6%)	0.03
ICI plus platinum-based chemotherapy	380 (48.6%)	17 (44.7%)	0.08
ICI plus antiangiogenic therapy	73 (9.3%)	4 (10.5%)	0.04
ICI plus chemotherapy and antiangiogenic therapy	45 (5.8%)	3 (7.9%)	0.08
Dual ICI therapy	46 (5.9%)	2 (5.3%)	0.03
Baseline autoimmune disease	33 (4.2%)	2 (5.3%)	0.05
Baseline systemic corticosteroid exposure	57 (7.3%)	4 (10.5%)	0.11
Acute infection during Day-21 window	78 (10.0%)	5 (13.2%)	0.10

ECOG, Eastern Cooperative Oncology Group; ICI, immune checkpoint inhibitor.

### 3.2 Baseline characteristics and ICI-associated myasthenia gravis phenotype

The Day-21 landmark cohort included 761 patients without post-Day-21 ICI-associated myasthenia gravis and 21 patients with post-Day-21 ICI-associated myasthenia gravis. Compared with non-event patients, event patients were older and showed higher standardized mean differences for male sex, current or former smoking status, ECOG performance status ≥2, baseline autoimmune disease, acute infection during the Day-21 window, and PD-L1 inhibitor exposure category. Baseline systemic corticosteroid exposure was uncommon in both groups. Treatment-regimen categories were broadly similar between groups, with no single regimen category explaining the post-Day-21 event distribution. Baseline characteristics of the Day-21 landmark cohort are shown in **Table 4**.

**Table 4:** Baseline characteristics of the day-21 landmark cohort.

Characteristic	No post-day-21 ICI-MG, n = 761	Post-day-21 ICI-MG, n = 21	Standardized mean difference
Age, years	65.4 ± 9.2	68.1 ± 8.7	0.30
Male sex	513 (67.4%)	16 (76.2%)	0.20
Current or former smoker	519 (68.2%)	17 (81.0%)	0.29
Body mass index, kg/m²	23.1 ± 3.4	22.6 ± 3.6	0.14
ECOG performance status ≥2	118 (15.5%)	5 (23.8%)	0.21
Adenocarcinoma	463 (60.8%)	12 (57.1%)	0.07
Squamous-cell carcinoma	244 (32.1%)	8 (38.1%)	0.13
Stage IV or recurrent disease	623 (81.9%)	18 (85.7%)	0.10
First-line ICI therapy	448 (58.9%)	13 (61.9%)	0.06
PD-L1 TPS ≥50%	191 (25.1%)	7 (33.3%)	0.18
EGFR mutation	86 (11.3%)	2 (9.5%)	0.06
ALK rearrangement	22 (2.9%)	1 (4.8%)	0.10
Liver metastasis	197 (25.9%)	6 (28.6%)	0.06
Brain metastasis	143 (18.8%)	5 (23.8%)	0.12
PD-1 inhibitor	501 (65.8%)	15 (71.4%)	0.12
PD-L1 inhibitor	216 (28.4%)	4 (19.0%)	0.22
PD-1/PD-L1 plus CTLA-4	44 (5.8%)	2 (9.5%)	0.14
ICI monotherapy	231 (30.4%)	7 (33.3%)	0.07
ICI plus chemotherapy	371 (48.8%)	9 (42.9%)	0.12
ICI plus antiangiogenic therapy	71 (9.3%)	2 (9.5%)	0.01
ICI plus chemotherapy and antiangiogenic therapy	44 (5.8%)	1 (4.8%)	0.05
Dual ICI therapy	44 (5.8%)	2 (9.5%)	0.14
Baseline autoimmune disease	31 (4.1%)	2 (9.5%)	0.22
Baseline systemic corticosteroid exposure	55 (7.2%)	2 (9.5%)	0.08
Acute infection during Day-21 window	74 (9.7%)	4 (19.0%)	0.27

ALK, anaplastic lymphoma kinase; CTLA-4, cytotoxic T-lymphocyte-associated protein 4; ECOG, Eastern Cooperative Oncology Group; EGFR, epidermal growth factor receptor; ICI, immune checkpoint inhibitor; ICI-MG, immune checkpoint inhibitor-associated myasthenia gravis; PD-1, programmed cell death protein 1; PD-L1, programmed death-ligand 1; TPS, tumor proportion score.

Among the 27 patients with ICI-associated myasthenia gravis in the baseline cohort, 18 were adjudicated as definite cases and 9 as probable cases. Among 64 trigger-positive suspected neuromuscular immune-related adverse-event records, initial agreement between the two independent reviewers was observed in 59 records, corresponding to 92.2% agreement and a Cohen κ of 0.84. The full two-reviewer agreement matrix is shown in **Table 5**. After resolution by the third reviewer of discordant records, 27 patients were classified as definite or probable ICI-associated myasthenia gravis. Eight patients had isolated myasthenia gravis, 10 had myositis overlap, five had myocarditis overlap, and four had myocarditis–myositis–myasthenia gravis overlap. Any myositis component was present in 14 patients, and any myocarditis component was present in nine patients. Intensive care admission occurred in seven patients, ventilatory support in six patients, intravenous immunoglobulin or plasma exchange in 12 patients, and myasthenia gravis-related death in three patients. The phenotype and severity distribution are shown in **Table 6**.

**Table 5:** Two-reviewer agreement matrix for trigger-positive suspected cases.

Reviewer 1/reviewer 2	ICI-MG positive	ICI-MG negative	Total
ICI-MG positive	25	3	28
ICI-MG negative	2	34	36
Total	27	37	64

Initial agreement: 59/64, 92.2%; Cohen κ = 0.84. ICI-MG, immune checkpoint inhibitor-associated myasthenia gravis.

**Table 6:** Phenotype and severity of ICI-associated myasthenia gravis.

Parameter	Value
Variable	ICI-MG cohort, n = 27
Definite ICI-associated myasthenia gravis	18 (66.7%)
Probable ICI-associated myasthenia gravis	9 (33.3%)
MG alone	8 (29.6%)
MG with myositis	10 (37.0%)
MG with myocarditis	5 (18.5%)
Myocarditis–myositis–MG overlap	4 (14.8%)
Any myositis component	14 (51.9%)
Any myocarditis component	9 (33.3%)
Onset ≤21 days	6 (22.2%)
Onset ≤42 days	15 (55.6%)
Ocular symptoms	16 (59.3%)
Bulbar symptoms	13 (48.1%)
Respiratory muscle involvement	7 (25.9%)
Limb or axial weakness	15 (55.6%)
AChR antibody positive among tested	11/22 (50.0%)
MuSK antibody positive among tested	1/18 (5.6%)
Abnormal repetitive nerve stimulation or single-fiber electromyography among tested	9/16 (56.3%)
Creatine kinase >5 times upper reference limit	14 (51.9%)
Troponin above upper reference limit	9 (33.3%)
ICU admission	7 (25.9%)
Non-invasive or invasive ventilation	6 (22.2%)
IVIG or plasma exchange	12 (44.4%)
MG-related death	3 (11.1%)

AChR, acetylcholine receptor; ICI-MG, immune checkpoint inhibitor-associated myasthenia gravis; ICU, intensive care unit; IVIG, intravenous immunoglobulin; MG, myasthenia gravis; MuSK, muscle-specific kinase.

### 3.3 Early serum tumor-marker and immune-inflammatory dynamics

**Figure 3:**
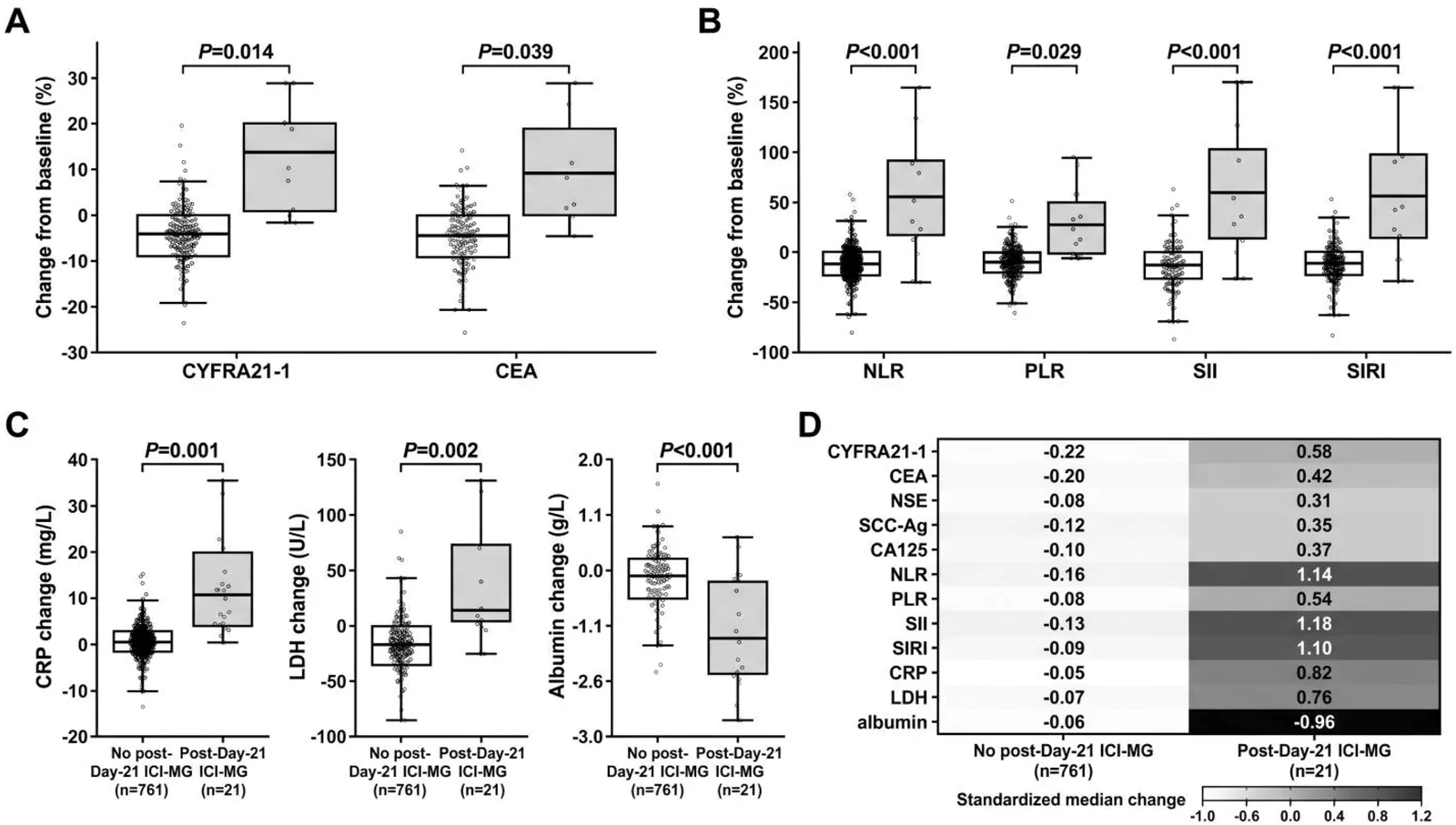
Day-21 biomarker changes by endpoint status. **(A)** Tumor markers. **(B)** Blood-cell inflammatory indices. **(C)** CRP, LDH, and albumin. **(D)** Standardized median-change heatmap. P values were calculated using the Wilcoxon rank-sum tests.

Baseline tumor-marker levels showed broad overlap between groups, whereas Day-21 changes separated more clearly. Median CYFRA21–1 changed by −8.9% in non-event patients and +26.7% in event patients, and median CEA changed by −10.6% and +18.4%, respectively. Immune-inflammatory changes showed a larger between-group shift: median NLR changed by −4.2% in non-event patients and +56.3% in event patients, median CRP changed by −0.7 mg/L and +15.8 mg/L, median LDH changed by −5 U/L and +51 U/L, and median albumin changed by −0.3 g/L and −3.4 g/L, respectively. Secondary inflammatory indices, including PLR, SII, and SIRI, showed the same direction of change. Day-21 biomarker missingness in the landmark cohort was 1.5% for complete blood count–derived indices, 4.7% for CEA, 5.1% for CYFRA21-1, 8.3% for NSE, 9.6% for SCC-Ag, 7.8% for CA125, 8.1% for CRP, 6.6% for LDH, and 1.4% for albumin. Day-21 biomarker dynamics are shown in **Figure 3** and **Table 7**.

**Table 7:** Day-21 biomarker dynamics in the landmark cohort.

Biomarker (unit)	Parameter	No post-day-21 ICI-MG *n* = 761	Post-day-21 ICI-MG *n* = 21	Descriptive P value
CYFRA21-1 (ng/mL)	Baseline	5.2 (2.4–11.6)	5.7 (2.8–12.9)	0.644
	Day-21	4.6 (2.0–10.1)	7.3 (3.9–17.8)	0.083
	Δ Change (%)	−8.9 (−32.4 to 14.6)	26.7 (−4.8 to 61.3)	0.014
CEA (ng/mL)	Baseline	11.9 (4.3–34.5)	13.2 (5.1–39.7)	0.706
	Day-21	10.4 (3.5–30.8)	16.9 (6.8–47.6)	0.126
	Δ Change (%)	−10.6 (−35.2 to 12.8)	18.4 (−9.1 to 54.9)	0.039
NSE (ng/mL)	Baseline	14.8 (10.7–21.6)	16.2 (11.9–25.4)	0.318
	Day-21	14.1 (10.2–20.3)	18.7 (12.6–29.1)	0.112
	Δ Change (%)	−3.6 (−18.9 to 13.4)	12.8 (−7.5 to 35.6)	0.082
SCC-Ag (ng/mL)	Baseline	1.8 (0.9–3.7)	2.1 (1.0–4.5)	0.401
	Day-21	1.6 (0.8–3.3)	2.5 (1.2–5.4)	0.138
	Δ Change (%)	−6.5 (−28.1 to 16.2)	14.9 (−8.2 to 42.1)	0.114
CA125 (U/mL)	Baseline	36.8 (18.2–75.6)	42.5 (19.7–86.4)	0.552
	Day-21	34.1 (16.9–70.2)	49.7 (24.5–101.3)	0.109
	Δ Change (%)	−5.4 (−25.7 to 18.8)	16.2 (−6.6 to 44.8)	0.073
NLR	Baseline	3.8 (2.6–5.5)	4.2 (2.8–6.1)	0.206
	Day-21	3.6 (2.3–5.4)	6.8 (4.5–10.7)	<0.001
	Δ Change (%)	−4.2 (−26.8 to 22.5)	56.3 (14.9 to 118.7)	<0.001
PLR	Baseline	172 (121–246)	184 (132–270)	0.337
	Day-21	166 (114–238)	224 (159–331)	0.044
	Δ Change (%)	−2.7 (−21.6 to 19.3)	24.5 (−2.1 to 58.4)	0.029
SII	Baseline	684 (421–1,104)	742 (466–1,216)	0.288
	Day-21	651 (389–1,067)	1,186 (721–1,836)	<0.001
	Δ Change (%)	−3.9 (−25.8 to 26.7)	61.4 (13.2 to 126.9)	<0.001
SIRI	Baseline	1.74 (0.98–2.88)	1.96 (1.11–3.35)	0.253
	Day-21	1.68 (0.91–2.76)	3.14 (1.78–5.41)	<0.001
	Δ Change (%)	−2.5 (−24.3 to 25.1)	58.6 (12.4 to 119.8)	<0.001
CRP (mg/L)	Baseline	8.5 (3.2–20.5)	11.8 (4.6–30.9)	0.084
	Day-21	7.8 (3.1–19.4)	28.6 (11.7–63.9)	0.002
	Δ Change (mg/L)	−0.7 (−6.4 to 6.9)	15.8 (3.2 to 39.6)	0.001
LDH (U/L)	Baseline	223 (183–279)	236 (196–315)	0.091
	Day-21	218 (177–272)	289 (229–361)	0.003
	Δ Change (U/L)	−5 (−38 to 29)	51 (8 to 104)	0.002
Albumin (g/L)	Baseline	39.4 (36.8–42.1)	39.2 (36.4–42.0)	0.822
	Day-21	39.1 (36.2–41.8)	35.7 (32.4–39.1)	<0.001
	Δ Change (g/L)	−0.3 (−1.8 to 1.1)	−3.4 (−5.6 to −1.2)	<0.001

CA125, carbohydrate antigen 125; CEA, carcinoembryonic antigen; CRP, C-reactive protein; CYFRA21-1, cytokeratin 19 fragment antigen 21-1; ICI-MG, immune checkpoint inhibitor-associated myasthenia gravis; LDH, lactate dehydrogenase; NLR, neutrophil-to-lymphocyte ratio; NSE, neuron-specific enolase; PLR, platelet-to-lymphocyte ratio; SCC-Ag, squamous cell carcinoma antigen; SII, systemic immune-inflammation index; SIRI, systemic inflammation response index.

### 3.4 Dynamic biomarker score and risk gradient

**Figure 4:**
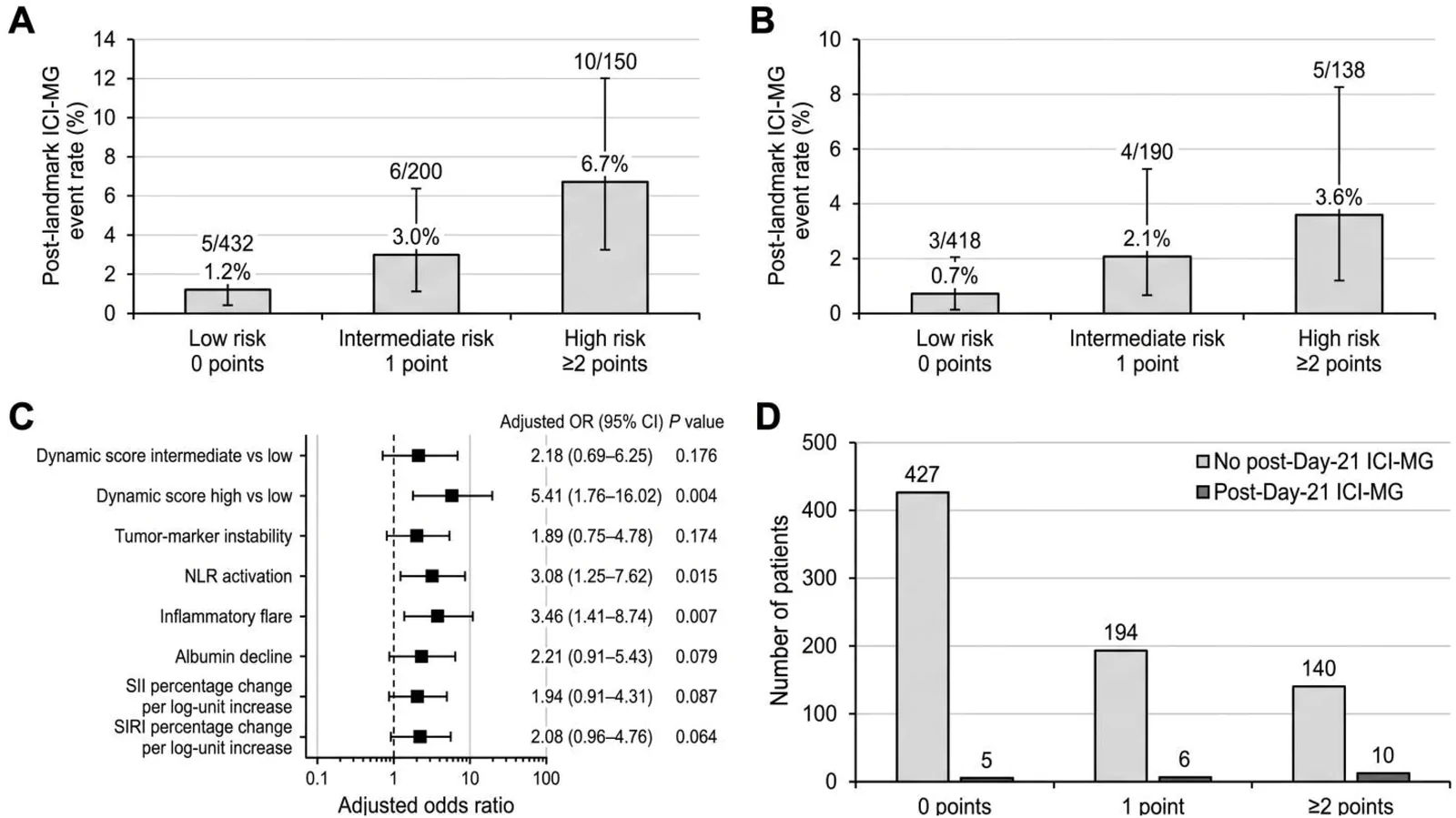
Dynamic biomarker score and component-level risk. **(A)** Post-Day-21 event rates across low-, intermediate-, and high-risk score strata. Error bars indicate 95% confidence intervals. **(B)** Post-Day-42 event rates across the same score strata. Error bars indicate 95% confidence intervals. **(C)** Forest plot of adjusted associations for score strata, score components, and secondary inflammatory indices. **(D)** Distribution of total Day-21 dynamic biomarker score by endpoint status.

The Day-21 dynamic biomarker score produced a graded endpoint distribution. Post-Day-21 ICI-associated myasthenia gravis occurred in five of 432 patients with 0 points, six of 200 patients with 1 point, and 10 of 150 patients with 2 or more points, corresponding to event rates of 1.2%, 3.0%, and 6.7%, respectively. The corresponding 95% confidence intervals were 0.4%–2.7%, 1.1%–6.4%, and 3.2%–12.0%. In the Day-42 landmark cohort, event rates were 0.7%, 2.1%, and 3.6% across the same score strata, with 95% confidence intervals of 0.1%–2.1%, 0.6%–5.3%, and 1.2%–8.3%, respectively. When each score component was examined in adjusted Firth logistic regression models, NLR activation and inflammatory flare showed the strongest individual associations with post-Day-21 ICI-associated myasthenia gravis. PLR, SII, and SIRI showed the same direction of Day-21 change as NLR but were retained as secondary biomarkers rather than added to the categorical score because they overlapped with complete blood count–derived inflammatory components. The combined score provided clearer risk separation than any single score component alone, as shown in **Figure 4** and **Tables 8**, **9**.

**Table 8:** Risk strata and post-landmark ICI-associated myasthenia gravis events.

Landmark cohort	Risk stratum	Definition	n	Events	Event rate	95% CI
Day 21	Low	0 points	432	5	1.2%	0.4%–2.7%
Day 21	Intermediate	1 point	200	6	3.0%	1.1%–6.4%
Day 21	High	≥2 points	150	10	6.7%	3.2%–12.0%
Day 42	Low	0 points	418	3	0.7%	0.1%–2.1%
Day 42	Intermediate	1 point	190	4	2.1%	0.6%–5.3%
Day 42	High	≥2 points	138	5	3.6%	1.2%–8.3%

CI, confidence interval.

**Table 9:** Adjusted Firth logistic regression for post-day-21 ICI-associated myasthenia gravis.

Variable	Adjusted odds ratio	95% CI	P value
Dynamic biomarker score: intermediate vs low	2.18	0.69–6.25	0.176
Dynamic biomarker score: high vs low	5.41	1.76–16.02	0.004
Age, per 10-year increase	1.28	0.82–2.05	0.286
Male sex	1.36	0.46–4.49	0.582
ECOG performance status ≥2	1.48	0.48–4.33	0.474
PD-1/PD-L1 plus CTLA-4 vs other ICI regimen	1.62	0.31–6.94	0.540
Baseline autoimmune disease	2.04	0.39–8.79	0.350
Baseline systemic corticosteroid exposure	1.12	0.21–4.65	0.881
Tumor-marker instability component	1.89	0.75–4.78	0.174
NLR activation component	3.08	1.25–7.62	0.015
Inflammatory flare component	3.46	1.41–8.74	0.007
Albumin decline component	2.21	0.91–5.43	0.079
SII percentage change, per log-unit increase	1.94	0.91–4.31	0.087
SIRI percentage change, per log-unit increase	2.08	0.96–4.76	0.064

The score strata and individual biomarker components were fitted in separate adjusted Firth models using the same clinical covariates. CI, confidence interval; CTLA-4, cytotoxic T-lymphocyte-associated protein 4; ECOG, Eastern Cooperative Oncology Group; ICI, immune checkpoint inhibitor; NLR, neutrophil-to-lymphocyte ratio; PD-1, programmed cell death protein 1; PD-L1, programmed death-ligand 1; SII, systemic immune-inflammation index; SIRI, systemic inflammation response index.

### 3.5 Prediction performance and internal validation

**Figure 5:**
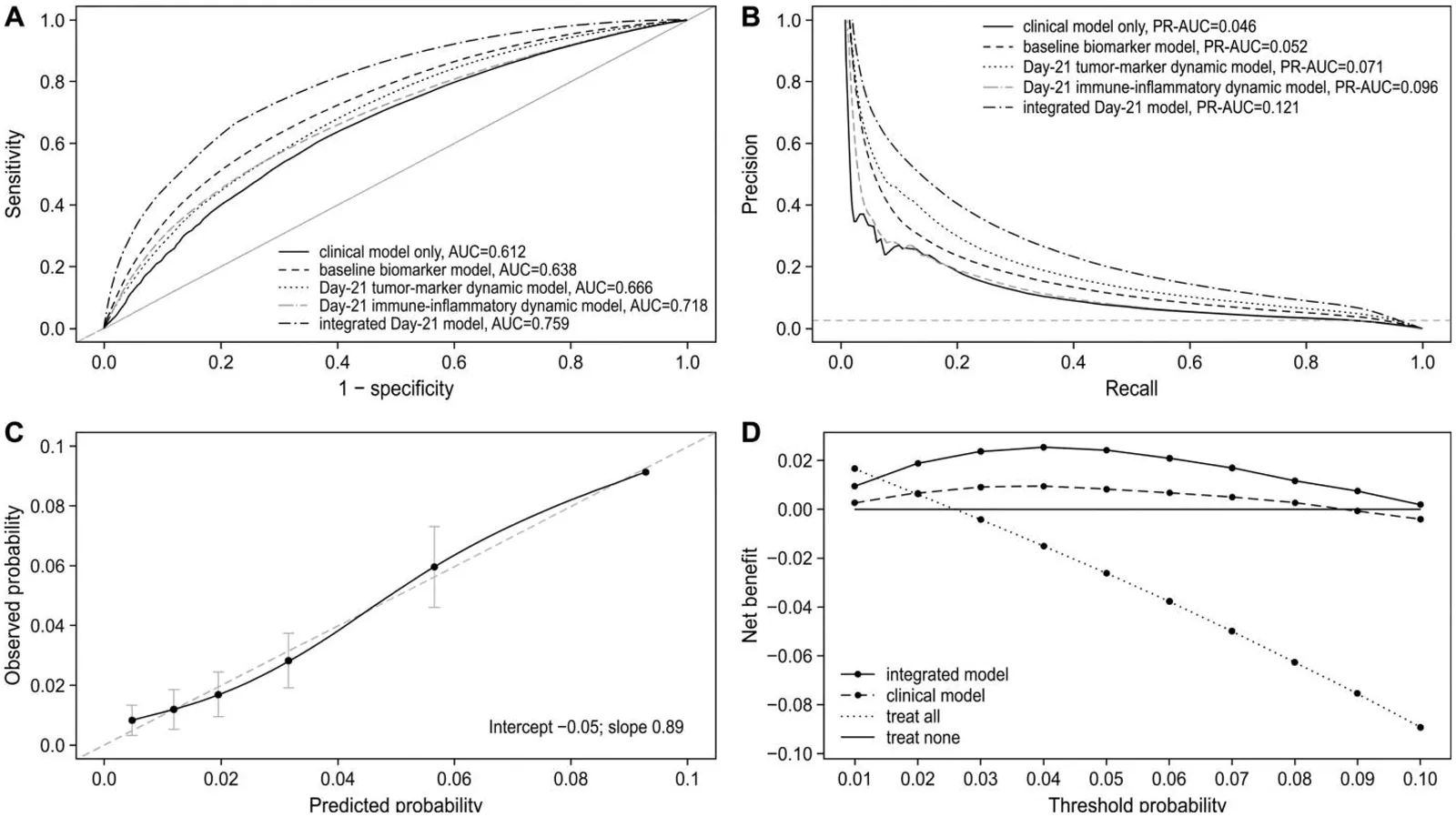
Prediction-model performance. **(A)** Receiver operating characteristic curves for the clinical, baseline biomarker, Day-21 tumor-marker dynamic, Day-21 immune-inflammatory dynamic, and integrated Day-21 models. **(B)** Precision–recall curves for the same models. **(C)** Calibration plot for the integrated Day-21 model. **(D)** Decision-curve analysis comparing the integrated model with the clinical model and default strategies.

The clinical model showed an AUC of 0.612 and a PR-AUC of 0.046 for post-Day-21 ICI-associated myasthenia gravis. The baseline biomarker model had an AUC of 0.638 and a PR-AUC of 0.052. Dynamic biomarker models performed better than baseline-only models, with an AUC of 0.666 for the Day-21 tumor-marker dynamic model and an AUC of 0.718 for the Day-21 immune-inflammatory dynamic model. The integrated Day-21 model had an apparent AUC of 0.759, a PR-AUC of 0.121, and a Brier score of 0.023. Bootstrap internal validation repeated score assignment, model fitting, and performance estimation within each resample. After 1,000 bootstrap resamples, the optimism-corrected integrated model retained an AUC of 0.731, a PR-AUC of 0.106, a calibration intercept of −0.04, and a calibration slope of 0.86. The complete-case estimate was similar to the multiply imputed estimate, with an AUC of 0.754 and a PR-AUC of 0.117. In the Day-42 secondary landmark model, the AUC was 0.746 and the Brier score was 0.016, with a lower PR-AUC and wider uncertainty because only 12 post-Day-42 events remained after landmark exclusion. Decision-curve analysis showed higher net benefit for the integrated model than for the clinical model across threshold probabilities from 2% to 8%. Model performance is shown in **Figure 5** and **Table 10**.

**Table 10:** Prediction performance for post-landmark ICI-associated myasthenia gravis.

Model	AUC	95% CI	PR-AUC	Brier score	Calibration intercept	Calibration slope
Clinical model only	0.612	0.494–0.719	0.046	0.026	−0.01	0.74
Baseline biomarker model	0.638	0.512–0.748	0.052	0.026	−0.02	0.78
Day-21 tumor-marker dynamic model	0.666	0.541–0.776	0.071	0.025	−0.03	0.81
Day-21 immune-inflammatory dynamic model	0.718	0.603–0.816	0.096	0.024	−0.03	0.84
Integrated Day-21 model	0.759	0.647–0.850	0.121	0.023	−0.05	0.89
Optimism-corrected integrated model	0.731	0.611–0.832	0.106	0.024	−0.04	0.86
Complete-case integrated model	0.754	0.638–0.846	0.117	0.023	−0.05	0.87
Day-42 secondary landmark model	0.746	0.586–0.866	0.093	0.016	−0.06	0.82

AUC, area under the receiver operating characteristic curve; CI, confidence interval; PR-AUC, area under the precision–recall curve.

### 3.6 Sensitivity and contextual analyses

**Figure 6:**
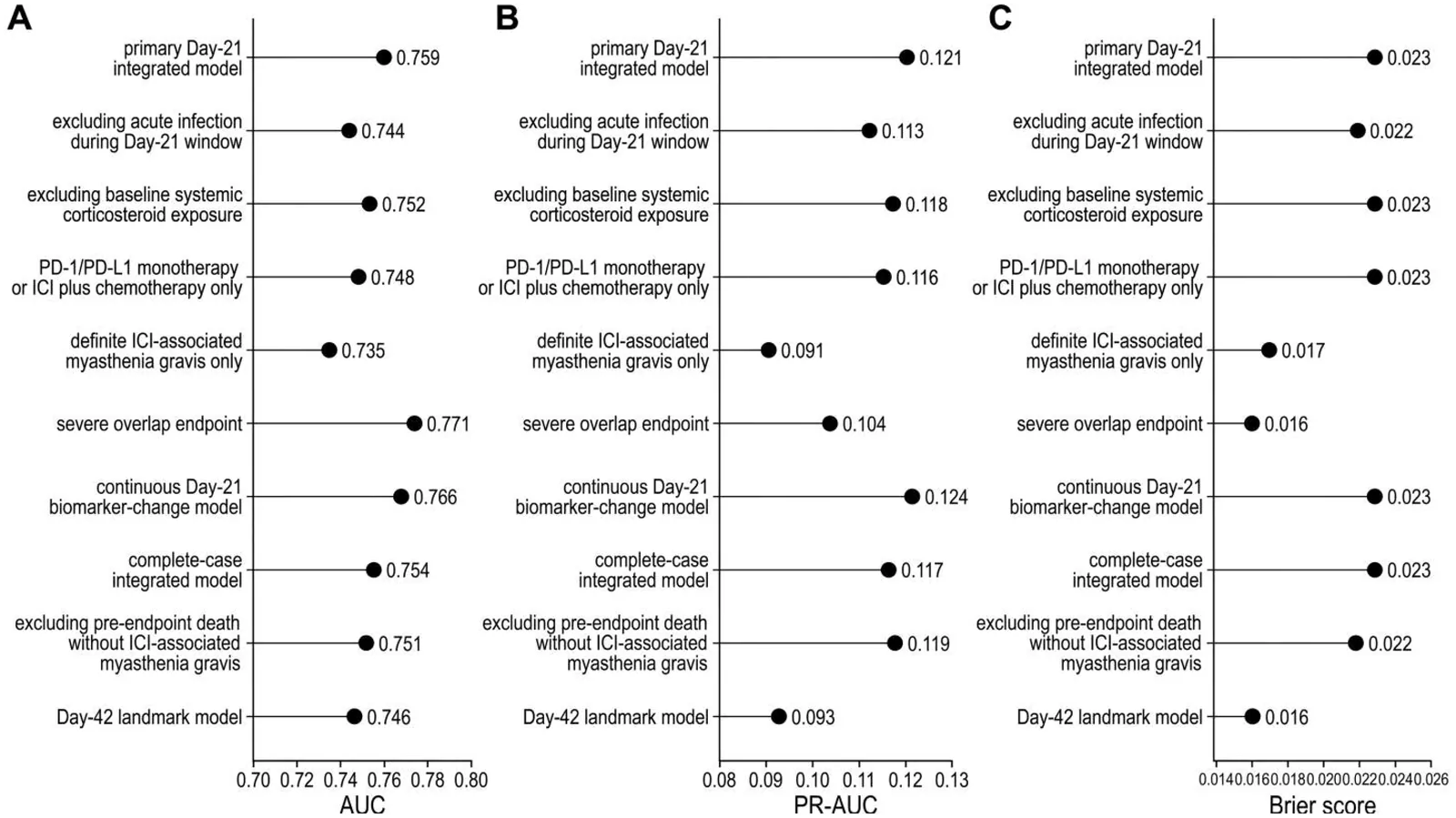
Sensitivity-analysis performance. **(A)** AUC values across prespecified sensitivity analyses of the integrated dynamic biomarker model. **(B)** PR-AUC values across the same analyses. **(C)** Brier scores across the same analyses.

The integrated model remained stable across prespecified sensitivity analyses. After excluding patients with acute infection during the Day-21 biomarker window, the AUC was 0.744 and the PR-AUC was 0.113. After excluding patients receiving baseline systemic corticosteroids, the AUC was 0.752 and the PR-AUC was 0.118. Restriction to PD-1/PD-L1 monotherapy or ICI plus chemotherapy yielded an AUC of 0.748 and a PR-AUC of 0.116. When the endpoint was restricted to definite ICI-associated myasthenia gravis, the AUC was 0.735 and the PR-AUC was 0.091. When the endpoint was changed to severe overlap syndrome, the AUC was 0.771 and the PR-AUC was 0.104. Replacing the categorical score with continuous Day-21 biomarker changes yielded an AUC of 0.766 and a PR-AUC of 0.124. The continuous-change model was numerically similar to the categorical score, while the categorical score offered simpler bedside interpretability. After excluding patients who died before ICI-associated myasthenia gravis without meeting the endpoint, the AUC was 0.751 and the PR-AUC was 0.119. Across all sensitivity analyses, AUC values ranged from 0.735 to 0.771 and PR-AUC values ranged from 0.091 to 0.124. Sensitivity-analysis performance is shown in **Figure 6** and **Table 11**.

**Table 11:** Sensitivity analyses of the integrated dynamic biomarker model.

Sensitivity analysis	Analysis cohort	Events	AUC	PR-AUC	Brier score
Primary Day-21 integrated model	782	21	0.759	0.121	0.023
Excluding acute infection during Day-21 window	704	17	0.744	0.113	0.022
Excluding baseline systemic corticosteroid exposure	725	19	0.752	0.118	0.023
PD-1/PD-L1 monotherapy or ICI plus chemotherapy only	716	19	0.748	0.116	0.023
Definite ICI-associated myasthenia gravis only	782	14	0.735	0.091	0.017
Severe overlap endpoint	782	14	0.771	0.104	0.016
Continuous Day-21 biomarker-change model	782	21	0.766	0.124	0.023
Complete-case integrated model	742	20	0.754	0.117	0.023
Excluding pre-endpoint death without ICI-associated myasthenia gravis	771	21	0.751	0.119	0.022
Day-42 landmark model	746	12	0.746	0.093	0.016

AUC, area under the receiver operating characteristic curve; ICI, immune checkpoint inhibitor; PR-AUC, area under the precision–recall curve.

Threshold-perturbation analyses showed that the score gradient was not dependent on a single narrow cutoff set. Across lenient, primary, and stricter threshold combinations, the high-risk stratum retained higher post-Day-21 event rates than the low-risk stratum, and model AUC values remained within a narrow range (**Table 12**).

**Table 12:** Threshold-perturbation analysis of the Day-21 dynamic biomarker score.

Threshold set	Main threshold changes	High-risk group, n	High-risk events	High-risk event rate	AUC	PR-AUC
Primary thresholds	CYFRA21-1 + 20%; CEA + 25%; NLR ≥5.0 or +50%; CRP + 10 mg/L or LDH + 40 U/L; albumin −3 g/L	150	10	6.7%	0.759	0.121
Lenient thresholds	CYFRA21-1 + 15%; CEA + 20%; NLR ≥4.0 or +30%; CRP + 5 mg/L or LDH + 30 U/L; albumin −2 g/L	236	14	5.9%	0.741	0.112
Stricter thresholds	CYFRA21-1 + 25%; CEA + 30%; NLR ≥6.0 or +70%; CRP + 15 mg/L or LDH + 50 U/L; albumin −4 g/L	96	8	8.3%	0.751	0.116
Tumor-marker thresholds varied only	CYFRA21-1 + 15% to +25%; CEA + 20% to +30%	137–166	9–11	6.3%–7.0%	0.754–0.762	0.118–0.124
Inflammatory thresholds varied only	NLR, CRP, and LDH varied across predefined ranges	129–178	9–12	6.0%–7.5%	0.746–0.766	0.115–0.126
Albumin threshold varied only	Albumin decline −2 to −4 g/L	141–163	9–11	6.2%–7.1%	0.752–0.761	0.117–0.123

AUC, area under the receiver operating characteristic curve; CEA, carcinoembryonic antigen; CRP, C-reactive protein; CYFRA21-1, cytokeratin 19 fragment antigen 21-1; LDH, lactate dehydrogenase; NLR, neutrophil-to-lymphocyte ratio; PR-AUC, area under the precision–recall curve.

Exploratory contextual analyses showed that high Day-21 risk scores were accompanied by numerically higher rates of progressive disease and non-myasthenic immune-related adverse events, but these patterns did not eliminate the graded ICI-associated myasthenia gravis risk signal (**Table 13**).

**Table 13:** Tumor response and non-myasthenic immune-related adverse events by Day-21 risk stratum.

Variable	Low risk, n = 432	Intermediate risk, n = 200	High risk, n = 150
Complete response	4 (0.9%)	1 (0.5%)	0 (0.0%)
Partial response	142 (32.9%)	58 (29.0%)	35 (23.3%)
Stable disease	169 (39.1%)	76 (38.0%)	55 (36.7%)
Progressive disease	72 (16.7%)	45 (22.5%)	44 (29.3%)
Not evaluable for response	45 (10.4%)	20 (10.0%)	16 (10.7%)
Any non-myasthenic immune-related adverse event	106 (24.5%)	59 (29.5%)	53 (35.3%)
Pneumonitis	32 (7.4%)	17 (8.5%)	16 (10.7%)
Dermatologic toxicity	39 (9.0%)	19 (9.5%)	13 (8.7%)
Thyroiditis or thyroid dysfunction	28 (6.5%)	15 (7.5%)	12 (8.0%)
Hepatitis	15 (3.5%)	9 (4.5%)	11 (7.3%)
Colitis	8 (1.9%)	6 (3.0%)	5 (3.3%)
Myositis or myocarditis without ICI-MG	3 (0.7%)	2 (1.0%)	3 (2.0%)

Response categories are mutually exclusive. Non-myasthenic immune-related adverse-event categories are not mutually exclusive. Abbreviations: ICI-MG, immune checkpoint inhibitor-associated myasthenia gravis.

## 4 Discussion

This study evaluated whether early changes in routinely available serum tumor markers and immune-inflammatory biomarkers could identify patients with NSCLC at increased risk of ICI-associated myasthenia gravis. Three main findings were observed. First, ICI-associated myasthenia gravis was uncommon but clinically consequential: 27 of 826 patients developed definite or probable disease within 180 days, more than half of the events occurred within 42 days, and overlap with myositis or myocarditis accounted for a substantial proportion of cases. Second, baseline biomarker levels showed limited separation, whereas Day-21 changes in CYFRA21-1, CEA, NLR, CRP, LDH, and albumin more clearly distinguished patients who later developed ICI-associated myasthenia gravis. Third, a four-component dynamic score combining tumor-marker instability, NLR activation, CRP/LDH inflammatory flare, and albumin decline produced a graded risk pattern and improved discrimination beyond clinical variables alone. The final model showed moderate internal performance, so the score should be interpreted as an early risk-stratification signal rather than a diagnostic test or a standalone clinical decision tool.

The timing and severity profile in this cohort is consistent with previous descriptions of ICI-associated myasthenia gravis. Makarious et al. reported that this toxicity often appears within the first several weeks after treatment initiation and is not consistently explained by acetylcholine receptor antibody positivity (5). A systematic review of anti-PD-1-associated neuromuscular adverse events further showed that myasthenia gravis, myositis, and neuropathic syndromes can emerge rapidly and overlap clinically (29). Subsequent clinical experience suggested that unfavorable outcomes are more closely linked to severe or overlapping presentations than to ocular symptoms alone (30). Recent systematic evidence on myocarditis, myositis, and myasthenia gravis overlap also supports the view that this combined phenotype represents a high-risk immune-related toxicity pattern (31). In the present cohort, more than half of ICI-associated myasthenia gravis cases had a myositis or myocarditis component, supporting the need for early surveillance that includes neuromuscular and cardiac overlap features rather than ocular weakness alone.

The tumor-marker component of the score should be interpreted cautiously. CEA and CYFRA21–1 are not mechanistic markers of myasthenia gravis, and a rising level does not indicate neuromuscular autoimmunity by itself. Their value in this study is more likely to lie in their role as clinically accessible signals of an unstable tumor–host interaction during the first treatment cycle. Previous NSCLC studies have shown that CEA, CYFRA21-1, and NSE dynamics can help monitor response or prognosis during immunotherapy, and that early serum tumor-marker changes may carry information not fully captured by baseline values (32–34). The exploratory contextual analyses showed that higher Day-21 risk strata also had numerically higher rates of progressive disease and non-myasthenic immune-related adverse events, indicating that tumor-marker instability may reflect broader tumor response and immune activation patterns rather than a myasthenia-specific process. In the current analysis, the tumor-marker signal alone was weaker than the immune-inflammatory signal, but it contributed to risk separation when combined with NLR, CRP, LDH, and albumin.

The immune-inflammatory findings are more directly aligned with the clinical biology of ICI toxicity, although they are still not disease-specific. NLR activation, CRP/LDH flare, and albumin decline are broad indicators of systemic inflammatory stress. Prior studies in NSCLC have linked peripheral blood markers, including neutrophil-related indices, platelet-related indices, lymphocyte counts, and inflammatory biomarkers, with immune-related adverse events or severe toxicity during ICI therapy (35–37). More broadly, immune-related adverse events have been associated with survival outcomes in patients with advanced NSCLC treated with ICIs (38), while current clinical guidelines emphasize the prompt recognition and appropriate management of potentially severe immune-related toxicities (39). Pooled randomized-trial analyses have also reported an association between immune-related adverse events and ICI efficacy in NSCLC (40). The present analysis extends this concept to a more specific neuromuscular endpoint. However, because CRP, LDH, NLR, SII, and SIRI are acute-phase indicators, Day-21 elevations may partly reflect subclinical or undiagnosed neuromuscular, myositic, infectious, or inflammatory processes. The landmark design established temporal ordering but did not prove causal independence from early evolving toxicity. This interpretation is supported by the sensitivity analyses excluding acute infection and baseline systemic corticosteroid exposure, which showed similar but not definitive model performance.

The model performance was plausible for a rare event but should not be overstated. The integrated Day-21 model increased the AUC from 0.612 with clinical variables alone to 0.759, with an optimism-corrected AUC of 0.731. The PR-AUC remained modest because the endpoint incidence was low. The continuous-change model performed similarly to the categorical score, but the threshold-based score was retained because it provides a simpler and more reproducible bedside risk-stratification approach. The high-score group had a higher event rate, but events still occurred in the low-score group; therefore, the score should not be used to rule out ICI-associated myasthenia gravis. Its more appropriate use is to intensify early symptom review, including ocular, bulbar, respiratory, myositic, and cardiac features, and to lower the threshold for CK, troponin, neurology consultation, or cardiology consultation when clinically indicated. The weaker Day-42 analysis likely reflected the smaller number of remaining post-Day-42 events after early cases were excluded by the landmark design.

Several limitations should be considered. This was a retrospective single-center study, so laboratory timing, diagnostic intensity, treatment selection, and consultation thresholds were not fully standardized. The number of ICI-associated myasthenia gravis events was limited, especially after landmark exclusion, which constrained the number of predictors and required bias-reduced modeling. Confirmatory tests, including antibody testing and electrophysiology, were not uniformly performed, and probable cases may have included some misclassification despite blinded adjudication. Tumor-marker assays, infection status, steroid exposure, treatment combinations, tumor response, and other immune-related adverse events may have introduced residual confounding. Although bootstrap validation was performed, no independent external or temporal validation cohort was available. The score should therefore be regarded as hypothesis-generating until validated in larger multicenter cohorts with harmonized neuromuscular and cardiac screening pathways.

## 5 Conclusion

Early dynamic changes in routine serum tumor markers and immune-inflammatory biomarkers were associated with subsequent ICI-associated myasthenia gravis in patients with NSCLC. A Day-21 score combining CYFRA21-1/CEA instability, NLR activation, CRP/LDH flare, and albumin decline identified a higher-risk subgroup and improved prediction beyond clinical variables alone. The model provides a noninvasive early risk-stratification signal rather than a diagnostic test, and external validation is required before its clinical implementation. Its main potential value is to support earlier recognition of ocular, bulbar, respiratory, myositic, and cardiac overlap features during the first weeks of ICI therapy.

## Data Availability

The original contributions presented in the study are included in the article/supplementary material. Further inquiries can be directed to the corresponding authors.

## References

[1] Bray F Laversanne M Sung H Ferlay J Siegel RL Soerjomataram I . Global cancer statistics 2022: GLOBOCAN estimates of incidence and mortality worldwide for 36 cancers in 185 countries. CA Cancer J Clin. (2024) 74:229–63. doi: 10.3322/caac.21834 38572751

[2] Thai AA Solomon BJ Sequist LV Gainor JF Heist RS . Lung cancer. Lancet. (2021) 398:535–54. doi: 10.1016/S0140-6736(21)00312-3 34273294

[3] Postow MA Sidlow R Hellmann MD . Immune-related adverse events associated with immune checkpoint blockade. N Engl J Med. (2018) 378:158–68. doi: 10.1056/NEJMra1703481 29320654

[4] Grangeon M Tomasini P Chaleat S Jeanson A Souquet-Bressand M Khobta N . Association between immune-related adverse events and efficacy of immune checkpoint inhibitors in non-small-cell lung cancer. Clin Lung Cancer. (2019) 20:201–7. doi: 10.1016/j.cllc.2018.10.002 30442524

[5] Makarious D Horwood K Coward JIG . Myasthenia gravis: an emerging toxicity of immune checkpoint inhibitors. Eur J Cancer. (2017) 82:128–36. doi: 10.1016/j.ejca.2017.05.041 28666240

[6] Johansen A Christensen SJ Scheie D Højgaard JLS Kondziella D . Neuromuscular adverse events associated with anti-PD-1 monoclonal antibodies: systematic review. Neurology. (2019) 92:663–74. doi: 10.1212/WNL.0000000000007235 30850443

[7] Shi J Tan Y Huang Y Li K Yan J Guan Y . Association between clinical factors and result of immune checkpoint inhibitor-related myasthenia gravis: a single-center experience and systematic review. Front Neurol. (2022) 13:858628. doi: 10.3389/fneur.2022.858628 35463153 PMC9022009

[8] Safa H Johnson DH Trinh VA Rodgers TE Lin H Suarez-Almazor ME . Immune checkpoint inhibitor-related myasthenia gravis: single-center experience and systematic review of the literature. J Immunother Cancer. (2019) 7:319. doi: 10.1186/s40425-019-0774-y 31753014 PMC6868691

[9] Pathak R Katel A Massarelli E Villaflor VM Sun V Salgia R . Immune checkpoint inhibitor-induced myocarditis with myositis/myasthenia gravis overlap syndrome: a systematic review. Oncologist. (2021) 26:1052–61. doi: 10.1002/onco.13931 34378270 PMC8649039

[10] Lang D Horner A Brehm E Akbari K Hergan B Lenz K . Early serum tumor marker dynamics predict progression-free and overall survival in single PD-1/PD-L1 inhibitor treated advanced NSCLC: a retrospective cohort study. Lung Cancer. (2019) 134:59–65. doi: 10.1016/j.lungcan.2019.05.033 31319996

[11] Pavan A Calvetti L Dal Maso A Attili I Del Bianco P Pasello G . Peripheral blood markers identify risk of immune-related toxicity in advanced non-small cell lung cancer treated with immune-checkpoint inhibitors. Oncologist. (2019) 24:1128–36. doi: 10.1634/theoncologist.2018-0563 31015312 PMC6693718

[12] von Elm E Altman DG Egger M Pocock SJ Gøtzsche PC Vandenbroucke JP . The Strengthening the Reporting of Observational Studies in Epidemiology statement: guidelines for reporting observational studies. Ann Intern Med. (2007) 147:573–7. doi: 10.7326/0003-4819-147-8-200710160-00010 17938396

[13] Collins GS Reitsma JB Altman DG Moons KGM . Transparent reporting of a multivariable prediction model for individual prognosis or diagnosis: the TRIPOD statement. Ann Intern Med. (2015) 162:55–63. doi: 10.7326/M14-0697 25560714

[14] Nicholson AG Tsao MS Beasley MB Borczuk AC Brambilla E Cooper WA . The 2021 WHO classification of lung tumors: impact of advances since 2015. J Thorac Oncol. (2022) 17:362–87. doi: 10.1016/j.jtho.2021.11.003 34808341

[15] Goldstraw P Chansky K Crowley J Rami-Porta R Asamura H Eberhardt WEE . The IASLC Lung Cancer Staging Project: proposals for revision of the TNM stage groupings in the forthcoming eighth edition of the TNM classification for lung cancer. J Thorac Oncol. (2016) 11:39–51. doi: 10.1016/j.jtho.2015.09.009 26762738

[16] Eisenhauer EA Therasse P Bogaerts J Schwartz LH Sargent D Ford R . New response evaluation criteria in solid tumours: revised RECIST guideline, version 1.1. Eur J Cancer. (2009) 45:228–47. doi: 10.1016/j.ejca.2008.10.026 19097774

[17] Zhang Z Yuan F Chen R Li Y Ma J Yan X . Dynamics of serum tumor markers can serve as a prognostic biomarker for Chinese advanced non-small cell lung cancer patients treated with immune checkpoint inhibitors. Front Immunol. (2020) 11:1173. doi: 10.3389/fimmu.2020.01173 32587591 PMC7298878

[18] Yang X Pan X Cheng X Zheng Y He X Liu Y . Dynamic monitoring of serum tumor markers as prognostic factors in patients with advanced non-small-cell lung cancer receiving first-line PD-1/PD-L1 inhibitors plus chemotherapy. Ther Adv Med Oncol. (2023) 15:17588359231206282. doi: 10.1177/17588359231206282 37920256 PMC10619361

[19] Kataoka N Katayama Y Yamada T Morimoto K Takeda T Okada A . CYFRA 21-1 predicts efficacy of combined chemoimmunotherapy in patients with advanced non-small cell lung cancer: a prospective observational study. Transl Lung Cancer Res. (2024) 13:1929–37. doi: 10.21037/tlcr-24-190 39263030 PMC11384485

[20] Peng L Wang Y Liu F Qiu X Zhang X Fang C . Peripheral blood markers predictive of outcome and immune-related adverse events in advanced non-small cell lung cancer treated with PD-1 inhibitors. Cancer Immunol Immunother. (2020) 69:1813–22. doi: 10.1007/s00262-020-02585-w 32350592 PMC7413896

[21] Sung MD Jang WS Kim HR Park JA Lim SM Kim HR . Prognostic value of baseline and early treatment response of neutrophil-lymphocyte ratio, C-reactive protein, and lactate dehydrogenase in non-small cell lung cancer patients undergoing immunotherapy. Transl Lung Cancer Res. (2023) 12:1506–16. doi: 10.21037/tlcr-23-7 37577328 PMC10413036

[22] National Cancer Institute . Common Terminology Criteria for Adverse Events (CTCAE), Version 5.0. Bethesda, MD: National Cancer Institute (2017).

[23] Jaretzki A III Barohn RJ Ernstoff RM Kaminski HJ Keesey JC Penn AS . Myasthenia gravis: recommendations for clinical research standards. Neurology. (2000) 55:16–23. doi: 10.1212/WNL.55.1.16 10891897

[24] Puzanov I Diab A Abdallah K Bingham CO Brogdon C Dadu R . Managing toxicities associated with immune checkpoint inhibitors: consensus recommendations from the Society for Immunotherapy of Cancer Toxicity Management Working Group. J Immunother Cancer. (2017) 5:95. doi: 10.1186/s40425-017-0300-z 29162153 PMC5697162

[25] Lyon AR López-Fernández T Couch LS Asteggiano R Aznar MC Bergler-Klein J . 2022 ESC Guidelines on cardio-oncology developed in collaboration with the European Hematology Association, the European Society for Therapeutic Radiology and Oncology and the International Cardio-Oncology Society. Eur Heart J. (2022) 43:4229–361. doi: 10.1093/eurheartj/ehac244 36017568

[26] Azur MJ Stuart EA Frangakis C Leaf PJ . Multiple imputation by chained equations: what is it and how does it work? Int J Methods Psychiatr Res. (2011) 20:40–9. doi: 10.1002/mpr.329 21499542 PMC3074241

[27] Heinze G Schemper M . A solution to the problem of separation in logistic regression. Stat Med. (2002) 21:2409–19. doi: 10.1002/sim.1047 12210625

[28] Vickers AJ Elkin EB . Decision curve analysis: a novel method for evaluating prediction models. Med Decis Making. (2006) 26:565–74. doi: 10.1177/0272989X06295361 17099194 PMC2577036

[29] Marco C Rubio-Infante N Leventhal J Linares J Cuatrecasas M Dalmau J . Myasthenia gravis induced by immune checkpoint inhibitors: an emerging neurotoxicity in neuro-oncology practice. J Clin Med. (2023) 12:130. doi: 10.3390/jcm12010130 36614930 PMC9821391

[30] Qin Y Zhou Y Yang Z Wang J Zhang H Li J . Prognosis of immune checkpoint inhibitor-induced myasthenia gravis: a single-center experience and systematic review. Front Neurol. (2024) 15:1372861. doi: 10.3389/fneur.2024.1372861 38633537 PMC11022771

[31] Lipe DN Qdaisat A Krishnamani PP Nguyen TD Chaftari P El Messiri N . Myocarditis, myositis, and myasthenia gravis overlap syndrome associated with immune checkpoint inhibitors: a systematic review. Diagnostics. (2024) 14:1794. doi: 10.3390/diagnostics14161794 39202282 PMC11353298

[32] Dal Bello MG Filiberti RA Alama A Orengo AM Mussap M Coco S . The role of CEA, CYFRA21-1 and NSE in monitoring tumor response to nivolumab in advanced non-small cell lung cancer patients. J Transl Med. (2019) 17:74. doi: 10.1186/s12967-019-1828-0 30849967 PMC6408784

[33] Dall’Olio FG Abbati F Facchinetti F Massucci M Melotti B Squadrilli A . CEA and CYFRA 21-1 as prognostic biomarkers and as tools for treatment monitoring in advanced NSCLC treated with immune checkpoint inhibitors. Ther Adv Med Oncol. (2020) 12:1758835920952994. doi: 10.1177/1758835920952994 33193825 PMC7607728

[34] Tang Y Cui Y Li Z Jiao Z Zhang Y He Y . Dynamics of early serum tumour markers and neutrophil-to-lymphocyte ratio predict response to PD-1/PD-L1 inhibitors in advanced non-small-cell lung cancer. Cancer Manag Res. (2021) 13:8241–55. doi: 10.2147/CMAR.S329963 34754244 PMC8572022

[35] Liu W Ma F Sun B Liu Y Tang H Luo J . Peripheral blood markers associated with immune-related adverse effects in patients who had advanced non-small cell lung cancer treated with PD-1 inhibitors. Cancer Manag Res. (2021) 13:765–71. doi: 10.2147/CMAR.S293200 33536784 PMC7850423

[36] Egami S Kawazoe H Hashimoto H Uozumi R Arami T Sakiyama N . Peripheral blood biomarkers predict immune-related adverse events in non-small cell lung cancer patients treated with pembrolizumab: a multicenter retrospective study. J Cancer. (2021) 12:2105–12. doi: 10.7150/jca.53242 33754009 PMC7974524

[37] Xu H Feng H Zhang W Wei F Zhou L Liu L . Prediction of immune-related adverse events in non-small cell lung cancer patients treated with immune checkpoint inhibitors based on clinical and hematological markers: real-world evidence. Exp Cell Res. (2022) 416:113157. doi: 10.1016/j.yexcr.2022.113157 35427598

[38] Ricciuti B Genova C De Giglio A Bassanelli M Dal Bello MG Metro G . Impact of immune-related adverse events on survival in patients with advanced non-small cell lung cancer treated with nivolumab: long-term outcomes from a multi-institutional analysis. J Cancer Res Clin Oncol. (2019) 145:479–85. doi: 10.1007/s00432-018-2805-3 30506406 PMC11810236

[39] Schneider BJ Naidoo J Santomasso BD Lacchetti C Adkins S Anadkat M . Management of immune-related adverse events in patients treated with immune checkpoint inhibitor therapy: ASCO guideline update. J Clin Oncol. (2021) 39:4073–126. doi: 10.1200/JCO.21.01440 34724392

[40] Socinski MA Jotte RM Cappuzzo F Nishio M Mok TSK Reck M . Association of immune-related adverse events with efficacy of atezolizumab in patients with non-small cell lung cancer: pooled analyses of the phase 3 IMpower130, IMpower132, and IMpower150 randomized clinical trials. JAMA Oncol. (2023) 9:527–35. doi: 10.1001/jamaoncol.2022.7711 36795388 PMC9936386

